# Involvement of a 1-Cys Peroxiredoxin in Bacterial Virulence

**DOI:** 10.1371/journal.ppat.1004442

**Published:** 2014-10-16

**Authors:** Gilberto Hideo Kaihami, José Roberto Fogaça de Almeida, Suelen Silvana dos Santos, Luis Eduardo Soares Netto, Sandro Rogério de Almeida, Regina Lúcia Baldini

**Affiliations:** 1 Departamento de Bioquímica, Instituto de Química, Universidade de São Paulo, São Paulo, Brazil; 2 Departamento de Análises Clínicas, Faculdade de Ciências Farmacêuticas, Universidade de São Paulo, São Paulo, Brazil; 3 Departamento de Genética e Biologia Evolutiva, Instituto de Biociências, Universidade de São Paulo, São Paulo, Brazil; Vanderbilt University, United States of America

## Abstract

The killing of bacterial pathogens by macrophages occurs via the oxidative burst and bacteria have evolved to overcome this challenge and survive, using several virulence and defense strategies, including antioxidant mechanisms. We show here that the 1-Cys peroxiredoxin LsfA from the opportunistic pathogen *Pseudomonas aeruginosa* is endowed with thiol-dependent peroxidase activity that protects the bacteria from H_2_O_2_ and that this protein is implicated in pathogenicity. LsfA belongs to the poorly studied Prx6 subfamily of peroxiredoxins. The function of these peroxiredoxins has not been characterized in bacteria, and their contribution to host-pathogen interactions remains unknown. Infection of macrophages with the *lsfA* mutant strains resulted in higher levels of the cytokine TNF-α production due to the activation of the NF-kB and MAPK pathways, that are partially inhibited by the wild-type *P. aeruginosa* strain. A redox fluorescent probe was more oxidized in the *lsfA* mutant-infected macrophages than it was in the macrophages infected with the wild-type strain, suggesting that the oxidative burst was overstimulated in the absence of LsfA. Although no differences in the phagocytosis rates were observed when macrophages were infected with wild-type and mutant bacteria in a gentamicin exclusion assay, a higher number of wild-type bacterial cells was found in the supernatant. This difference was not observed when macrophages were pre-treated with a NADPH oxidase inhibitor, confirming the role of LsfA in the bacterial resistance to ROS generated via NADPH oxidase. In an acute pneumonia model, mice infected with the mutant strains presented higher cytokine release in the lungs and increased activated neutrophil recruitment, with reduced bacterial burden and improved survival rates compared to mice infected with the wild-type bacteria. LsfA is the first bacterial 1-Cys Prx shown to modulate host immune responses and its characterization will allow a better understanding of the role of redox signaling in host-pathogen interactions.

## Author Summary

*Pseudomonas aeruginosa* is an important human pathogen that employs a vast arsenal of virulence factors and infects immunocompromised hosts, such as patients in intensive care units, causing pneumonia and other illnesses. Macrophages are cells in the first line of defense against pathogens in the lungs. After pathogen recognition, macrophages release pro-inflammatory cytokines to recruit other immune cells and employ a process known as oxidative burst to kill invading microbes. *P. aeruginosa* can counteract oxidative stress using antioxidant proteins, such as peroxiredoxins. We show here that LsfA, which belongs to the poorly characterized Prx6 subfamily of peroxiredoxins, is indeed endowed with a thiol-dependent activity that is required for full virulence. *In vitro* and *in vivo* infection models confirmed that LsfA peroxidase activity is required for the immunomodulation caused by *P. aeruginosa* and that its absence allows the host to overcome the infection. This study demonstrates for the first time the involvement of a bacterial Prx6 in virulence.

## Introduction

*Pseudomonas aeruginosa* is a ubiquitous Gram-negative bacterium that can cause diseases in several hosts [1]. *P. aeruginosa* acute infections are one of the major problems in immunodeficient subjects, burn victims and mechanical ventilation patients. Pulmonary infections caused by *P. aeruginosa*, including ventilator-associated pneumonia and chronic pulmonary infection in cystic fibrosis patients, are associated with high mortality rates, and chronic pulmonary infection impairs life quality and life expectancy of the infected individuals [2]–[4]. The high intrinsic resistance of *P. aeruginosa* to antibiotics adds to the difficulties of treating infections caused by this versatile opportunist [5].

Macrophages are the first line of defense in pulmonary infections and play a major role in the host response to *P. aeruginosa* infections. Pathogens are recognized by the immune system, which detects pathogen-associated molecular patterns (PAMPs) by the corresponding pattern recognition receptor. The activation of signaling cascades by the binding of PAMPs to Toll-like receptors (TLRs), except for TLR-3, depends on MyD88 and results in the activation of the NF-κB and MAPK pathways [6]; their activation leads to the production of cytokines, including TNF-α, IL-6 and IL-1. The role of TLR-4 and TLR-5 in triggering a protective immunity against *P. aeruginosa* has been shown *in vivo*; as mice lacking TLR-4/5 have increased susceptibility to pulmonary infections [7], [8].

After the recognition of bacteria by the TLRs in macrophages, the signaling cascade leads to the generation of reactive oxygen species (ROS) in a process known as the oxidative burst, which depends on NADPH oxidase [9]. The oxidative burst is bactericidal and can cause lipid, protein and DNA lesions, resulting in pathogen clearing. However, to overcome or prevent these lesions, pathogens have developed a complex detoxification system that includes superoxide dismutase, and catalase/peroxidases that have been extensively studied in several pathogens, including *P. aeruginosa* [10], [11].

Among hydroperoxide-reducing enzymes, the peroxiredoxins (Prxs) are considered cellular sensors due to their abundance and reactivity [12]. Prxs catalyze the reaction ROOH+2e^−^→ROH+H_2_O and reduce hydrogen peroxide, peroxynitrite and a wide range of organic hydroperoxide compounds [13]–[15]. Prxs are found in organisms belonging to all Domains of life, indicating their crucial physiological function, but their role in *P. aeruginosa* virulence remains uncharacterized.

Prxs are a large family of proteins that can be divided into six sub-groups with distinct amino acid sequences, but all contain the thioredoxin fold and the PXXT(S)XXC motif [16]. Among these six-subgroups, Prx enzymes can display 2-Cys Prx or 1-Cys-Prx mechanisms, depending on the number of cysteine residues involved in catalysis [16]. AhpC, a 2-Cys Prx, is involved in the virulence of *Helicobacter cinaedi* and *Staphylococcus aureus* [17], [18], but it does not seem to be a virulence determinant for other bacteria that have been analyzed [19]–[21].

The genome of the highly virulent *P. aeruginosa* strain PA14 contains at least eight genes that encode Prxs, including AhpC and Tpx. Ohr is another Cys-based peroxidase from *P. aeruginosa* that has been structurally and enzymatically characterized [22], but it is not required for virulence [23].

Among the six Prx sub-groups, Prx6 is the least well studied. The precise physiological roles of the Prx6 sub-group remain unknown, with few reports addressing their kinetics and structural functions; most of these reports were from studies of eukaryotes [24]–[29]. Thus far, all of the Prx6 proteins characterized display the 1-Cys Prx mechanism. Remarkably, although the bacterial Domain contains hundreds of Prx6 representatives [16], no characterization of their roles has been reported.

In the *P. aeruginosa* genome, only one gene coding for a putative Prx6 is present (PA14_19490 in PA14; PA3450 in PA01). It was named *lsfA* because its expression is up-regulated, together with a gene cluster coding for an ABC-transport system involved in organic sulfur uptake, in cells grown in low-sulfate medium [30]. However, there is no experimental evidence for the mechanism underlying the function of LsfA in this process. It has been suggested that LsfA up-regulation and AhpC expression in low sulfate conditions may be a response to the oxidative stress caused by the excess levels of reduced flavin nucleotides due to sulfonate utilization [31]. Several transcriptomic analyses have revealed that *lsfA* expression is up-regulated in other stressful conditions, including in the presence of sodium hypochlorite [32], a product of the macrophage oxidative burst. Proteomic analyses identified LsfA as differentially expressed during other stressful conditions. Three LsfA isoforms are induced by the superoxide-generating drug paraquat [33] and in *P. aeruginosa* biofilms [34]. In iron starvation conditions, *Pseudomonas putida* also showed increased levels of the LsfA ortholog protein [35]. Interestingly, indole treatment, which may mimic conditions of iron abundance, decreased *lsfA* expression as well as virulence-related traits [36]. A more recent report found that the oxidation responsive OxyR activator protein binds to the *lsfA* promoter region [37]; this observation supports the role of LsfA in the bacterial response to H_2_O_2_.

Here, we show that the antioxidant function of the bacterial 1-Cys Prx LsfA is important for *P. aeruginosa* virulence, both in a macrophage model *in vitro* and in an acute pneumonia model *in vivo*. This work reveals the role of this protein as a novel virulence factor that contributes to the *P. aeruginosa* arsenal against host defenses and allows it address other stresses in various environmental conditions.

## Results

### LsfA is an active peroxiredoxin and is important for the antioxidant response

**Figure 1:**
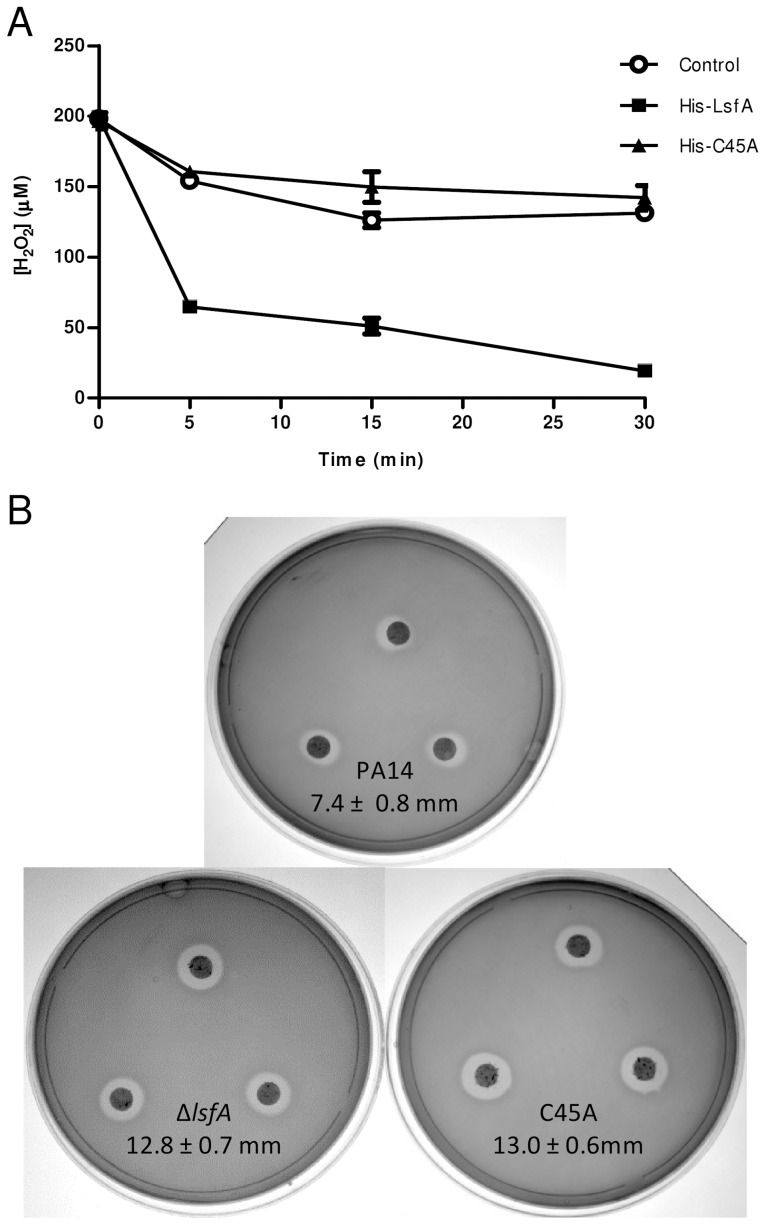
LsfA confers PA14 resistance to hydrogen peroxide and reduces it *in vitro*. (**A**) The recombinant proteins His-LsfA and His-C45A were incubated with 200 µM H_2_O_2_. The amount of H_2_O_2_ remaining in the reaction mixtures was determined by ferric-thiocyanate assays at the indicated time points. In a control assay, H_2_O_2_ was incubated with buffer without recombinant proteins. The data are the means ± SD from at least three independent experiments performed in triplicate. (**B**) Bacteria were grown to an OD_600 nm_ of 1 and spread on LB plates. A 6 mm filter disk containing 2.5% H_2_O_2_ was placed on top of the bacterial lawn, and the plates were incubated at 37°C for 16 hours. The data represent 1 of 5 independent experiments and the diameters of the inhibition haloes are shown as the means ± SD (mm).

A previous sequence alignment revealed that LsfA belongs to the Prx6 subfamily [16], with the Cys45 of LsfA as the putative peroxidasic cysteine (Fig. S1). To determine whether *P. aeruginosa* LsfA is indeed endowed with thiol-dependent peroxidase activity, the recombinant wild-type (His-LsfA) protein and a mutant protein without the putative catalytic cysteine (His-C45A) were expressed in *Escherichia coli* and purified by affinity chromatography. As predicted, H_2_O_2_ was reduced in the presence of wild-type His-LsfA but not when His-C45A was employed (Fig. 1A), showing that Cys45 is essential for catalysis and confirming that LsfA is an active 1-Cys Prx.

Because 2-Cys Prxs play an important role in bacterial protection against H_2_O_2_, *tert*-butyl hydroperoxide and paraquat [13], [14], we assessed whether LsfA was also important for *P. aeruginosa* resistance to such oxidants. To test this hypothesis, an in-frame deletion mutant strain (Δ*lsfA*) and a strain with a point mutation in the catalytic cysteine (C45A) were constructed. Both mutant strains grow like wild-type in minimal medium and in biofilms, excluding any growth defects (Fig. S2). The wild-type, Δ*lsfA* and C45A strains were tested using disk diffusion halo assays in the presence of oxidants. Larger inhibition haloes due to H_2_O_2_ were observed for both mutant strains (12.8±0.7 mm for Δ*lsfA*, 13.0±0.6 mm for C45A) compared with PA14 (7.4±0.8 mm for PA14) (Fig. 1B), indicating that LsfA is important for oxidative stress resistance in *P. aeruginosa* and confirming that the C45 catalytic cysteine is essential for LsfA activity. Complementation of the mutant strains with a copy of the *lsfA* gene in a plasmid restored the wild-type phenotype (Fig. S3), confirming that the larger haloes were due to a lack of LsfA activity. The *lsfA* mutants did not demonstrate increased sensitivity to paraquat or *tert*-butyl hydroperoxide (data not shown), which may reflect a compensatory effect of other Prxs and/or Ohr.

### LsfA is required for the inhibition of macrophage activation by *P. aeruginosa*

**Figure 2:**
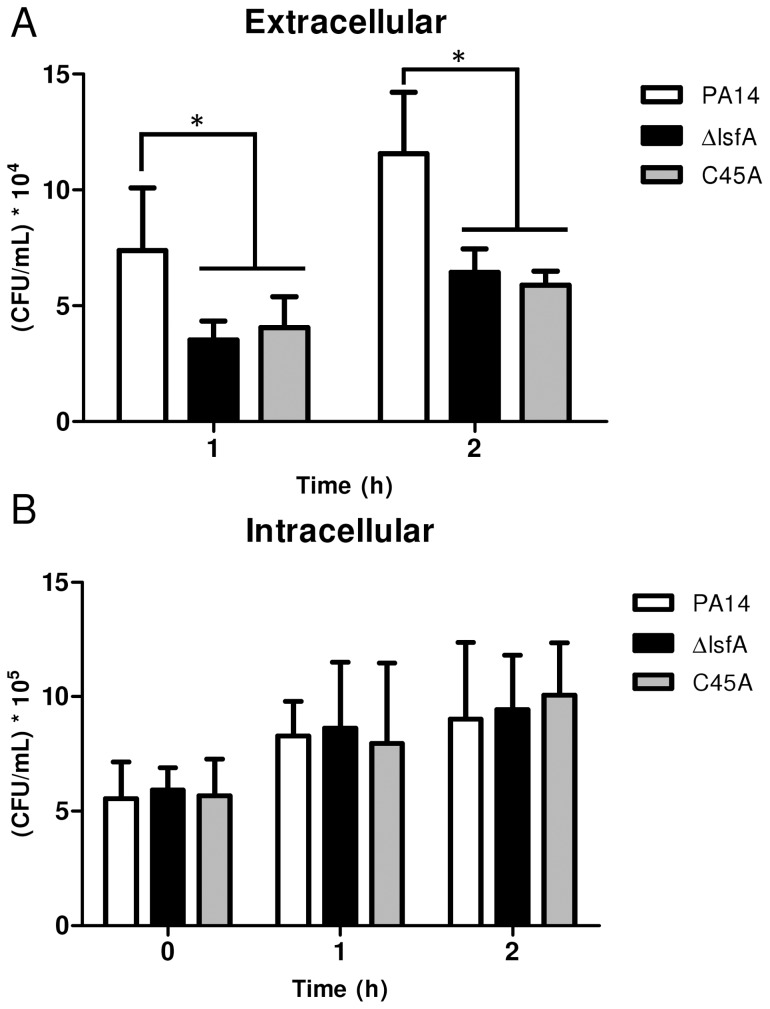
The *lsfA* mutants are phagocytosed at the same rate as PA14, but do not survive as well as PA14 in the cultures supernatants. J774 macrophages were incubated with *P. aeruginosa* PA14 or the Δ*lsfA* or C45A mutants at an MOI of 10. (**A**) At the indicated time points, the supernatants were collected and diluted, and the number of CFU was determined. (**B**) The cells were washed with PBS and R-10 containing 200 µg/mL gentamicin was added to the wells. At the indicated time points, the macrophages were lysed with Triton X-100. The released bacteria were diluted, and number of CFU was determined. The data shown are the means ± SD from at least three independent experiments performed in triplicate. *, p<0.05.

Because macrophages and neutrophils produce ROS and reactive nitrogen species in response to pathogens, bacterial antioxidant systems are important mechanisms that allow bacteria to overcome the deleterious effects of oxidative lesions and to survive during infection. To assess whether the *P. aeruginosa* LsfA is related to virulence, an *in vitro* model of infection in J774 macrophages was used. The macrophages were infected with PA14 and the *lsfA* mutants, and incubated for 1 hour prior to the gentamicin addition. At regular time points, the number of bacterial cells remaining in the culture supernatants was assessed (Fig. 2A), the macrophages were washed and lysed, and the released bacteria were counted (Fig. 2B). The phagocytosed bacteria counts were similar for all strains and showed a slight increase over time (Fig. 2B), suggesting that all of the strains were internalized by macrophages at the same extent. However, for the *lsfA* mutants, a 2-fold reduction in extracellular colony-forming units (CFUs) compared with the wild-type PA14 was observed (Fig. 2A). Thus, the peroxidase activity of LsfA contributes to bacterial viability in the presence of macrophages. The number of remaining macrophages at the end of the assay, assessed by a LDH release assay, was similar for all bacterial strains tested, showing that LsfA does not alter cytotoxicity (Fig. S4).

**Figure 3:**
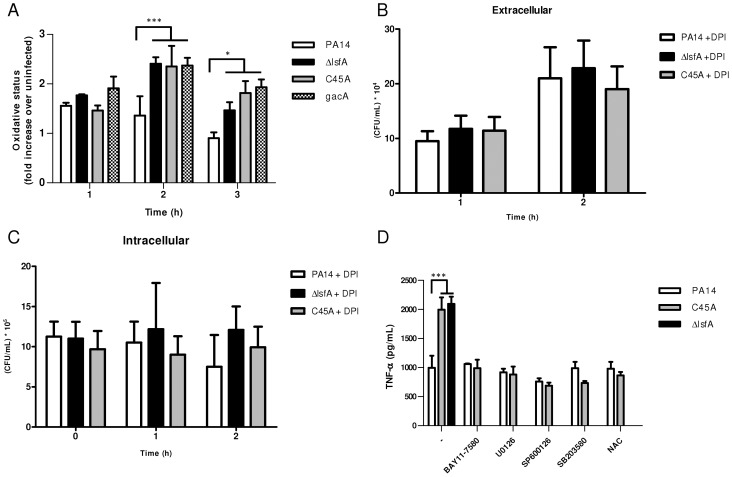
LsfA downregulates macrophages oxidative state that protected the bacteria against NADPH oxidase-generated ROS, and inhibits TNF-α production via the MAPK and NF-κB pathways. (**A**) J774 cells were infected with PA14 or the Δ*lsfA*, C45A, or *gacA* mutants. After 1, 2 or 3 hours, the macrophages were washed with PBS and incubated for 15 min with H_2_DCFDA. Fluorescence was analyzed by FACS. (**B**) Macrophages were treated with DPI prior to infection. The supernatants were collected and diluted at the indicated time points, and the number of CFU was determined. (**C**) Cells were washed with PBS and R-10 containing 200 µg/mL gentamicin was added to the wells. At the indicated time points, the macrophages were lysed with Triton X-100. The released bacteria were diluted and number of CFU was determined (**D**) Macrophages were infected with PA14 or the *lsfA* mutants in the absence (-) or the presence of inhibitors of NF-κB (BAY11-7580), ERK (U0126), JNK (SP600125) and p38 (SB2035-80) or the antioxidant *N-*acetylcysteine (NAC). After 3 hours of infection, the supernatants were recovered, and TNF-α secretion was determined by ELISA. Data are the means ± SD from three independent experiments performed in triplicate. *, p<0.05; ***, p<0.001.

The increased survival of PA14 compared with the *lsfA* mutants may reflect a change in the oxidative status of the macrophages in addition to an improved bacterial response to the oxidative burst. To address this hypothesis, macrophages were incubated with wild-type or mutant strains, and at different times, the cells were washed and incubated with 2′,7′-dichlorodihydrofluorescein diacetate (H_2_DCFDA), a probe that can sense shifts in the cellular redox state. After 3 hours of treatment, an increase in the intracellular oxidative status in macrophages incubated with the *lsfA* mutant strains was observed, in comparison with macrophages infected with PA14 (Fig. 3A). This result suggests that LsfA participates in the response of *P. aeruginosa* to the oxidative insult caused by the macrophages, most likely due to its peroxidase activity. Because LsfA affects the macrophage redox status, it can also impact virulence by subverting the host signaling pathways [38]. The mutant for *gacA*, which encodes a protein that plays a role in *P. aeruginosa* pathogenicity, has been extensively studied [39] and caused a similar increase in the macrophage oxidative state (Fig. 3A), suggesting that LsfA may indirectly inhibit the oxidative burst.

To evaluate the role of ROS generated by NADPH oxidase in the clearance of *lsfA* mutant strains, a gentamicin exclusion assay was performed again with macrophages, now pre-treated with DPI, a NADPH oxidase inhibitor. The CFU number corresponding to the bacteria that survive inside the DPI-treated macrophages is higher than in untreated ones (Fig. 2B and 3B), suggesting that they are no longer able to kill the bacteria. Moreover, the number of PA14 or the mutant cells under DPI treatment were similar both intra and extracellular, suggesting that LsfA counteracted NADPH oxidase activity during PA14 evasion from the macrophages (Fig. 3B and C). To understand whether *P. aeruginosa* LsfA modulates macrophage activation, J774 cells were infected with PA14 or the *lsfA* mutant strains. The supernatants were recovered after 3 hours, and cytokine levels were measured. Macrophages infected with the *lsfA* mutants secreted more TNF-α than macrophages infected with PA14 (Fig. 3D). This result suggests that the LsfA function decreases macrophage activation and, thus, its oxidative state because the oxidative burst is less pronounced when macrophages are infected with the wild-type strain.

C45A-infected macrophages treated with the thiol-reductant *N*-acetylcysteine (NAC) showed a reduced oxidative state (Fig. S5) and lower TNF-α production, similar to that of PA14-infected macrophages either in the presence or absence of NAC (Fig. 3D). Both NAC and LsfA may prevent macrophage activation by decreasing the oxidative state of phagocyte and thereby subverting signaling pathways involved in the immunological response.

Indeed, ROS can act as signaling molecules that lead to NF-κB and MAPK activation and increased cytokine production [40]. To determine which signaling pathways are up-regulated when the macrophages are infected with *lsfA* mutant strains, specific inhibitors of NF-κB or the MAPKs ERK1/2, JNK or p38 were used. In the presence of these inhibitors, no differences in TNF-α production were observed when macrophages were infected with either PA14 or C45A strains (Fig. 3D). These data suggest that LsfA peroxidase activity in PA14 decreases the activation of the NF-κB and MAPK pathways, and this decreased activation is reflected in the lower oxidative state of macrophages.

### LsfA is essential for *P. aeruginosa* full virulence *in vivo*

**Figure 4:**
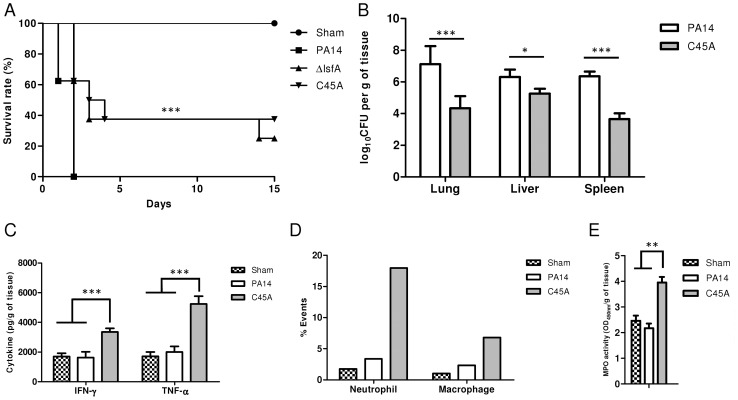
LsfA plays an important role in virulence in an acute pneumonia model. (**A**) BALB/c mice were infected i.t. with 2×10^6^ bacteria per mouse with wild-type strain PA14 or the Δ*lsfA* or C45A mutants (n = 8 per group). Control mice were inoculated with PBS (n = 3) and the survival of the mice was followed for the course of the experiment. (**B**) At 24 hours post-infection, three animals were sacrificed, the organs were macerated, and the bacterial CFU were enumerated. (**C**) The cytokines in the lungs were determined by ELISA. (**D**) Mice were infected with PA14 or the C45A mutant. At 24 hours post-infection, the animals were euthanized, and the lungs were macerated. The cell suspensions were labeled for macrophages (F4/80^+^; CD11b^+^) or neutrophils (Ly6G/Ly6C^+^; F4/80^−^) and analyzed by FACS. (**E**) Mice were infected with PA14 or the C45A mutant. At 24 hours post-infection, the animals were euthanized, and the lungs were macerated, the cells were lysed by sonication and the suspension was centrifuged. The resulting supernatant was used for MPO activity assay with TMB., and OD was measured at 450 nm. The data are representative of three (**B**, **C**) or two (**D**, **E**). independent experiments independent experiments *, p<0.05; **, p<0.01; ***, p<0.001.

Because LsfA is required for virulence in macrophages *in vitro*, the next step was to ascertain whether this 1-Cys Prx belonging to Prx6 group was also relevant in an acute pneumonia model in mice. Although all mice infected intratracheally (i.t.) with PA14 were dead 48 hours after infection, mice infected with *lsfA* strains had higher survival rates, with 37.5% of animals still alive after 13 days (Fig. 4A). After 60 days, surviving mice that had previously been infected with Δ*lsfA* (37.5%) and C45A (25%) seemed healthy, indicating that the infection had resolved or became chronic. In conclusion, the requirement of LsfA activity for *P. aeruginosa* virulence was confirmed.

*P. aeruginosa* introduced i.t. into mice disseminates quickly and affects other organs, leading to death [8]. Bacterial loads were assessed in the primary site of infection as well as in secondary organs (liver and spleen) as an indication of sepsis. Lungs, spleen and liver were recovered 24 hours p.i., and bacterial counts were evaluated. Animals infected with the C45A mutant strain showed a reduced bacterial burden in all organs analyzed compared with PA14-infected animals (Fig. 4B). This result indicates an improved bacterial resolution in mice infected with the C45A strain and demonstrates the relevance of the 1-Cys Prxs to virulence.

To ascertain whether LsfA would impact cytokine production *in vivo*, thus potentially decreasing the immune system activation and neutrophil recruitment to the infection site, the TNF-α and IFN-γ levels in the lungs of infected animals were determined by ELISA at 24 hours p.i. The wild-type strain induced local immunosuppression, with TNF-α and IFN-γ release similar to that observed in the control mice (Fig. 4C). However, mice infected with C45A released higher levels of these cytokines (Fig. 4C). No differences were observed in the anti-inflammatory cytokine IL-10 in mice infected with either PA14 or the *lsfA* mutant strain compared to the control (Fig. S6). In addition, C45A-infected mice showed an increased recruitment of neutrophils (Ly6G/Ly6C^+^; F4/80^−^) and macrophages (F4/80^+^; CD11b^+^) to the lungs compared with the controls (Fig. 4D). To determine if the recruited neutrophils were activated, myeloperoxidase (MPO) activity was present in all treatments, but it was higher when mice were infected with the C45A mutant strain (Fig. 4E), suggesting that thiol peroxidase activity is also involved in neutrophil activation. This set of data confirms that a better immune response that is reflected in the higher survival of the *lsfA* mutant-infected animals compared to those infected with PA14.

## Discussion

In this study, we show for the first time the relevance of the 1-Cys Prx LsfA in bacterial virulence. LsfA, a Prx from *P. aeruginosa*, is the first protein in the Prx6 sub-group to be connected to pathogenicity. This protein, LsfA, has orthologues in other pathogens, including *Burkholderia* and *Bordetella*, suggesting that this host-pathogen interaction may be present in other bacteria. Biochemical analysis demonstrated that LsfA has peroxidase activity that depends on the catalytic cysteine (C45). Inactivating this peroxidase activity makes *lsfA* mutant cells more sensitive than wild-type cells to hydrogen peroxide but not to organic peroxides, including *tert*-butyl-hydroperoxide. In addition, LsfA may also be endowed with other enzymatic activity because LsfA displays a conserved lipase motif (GDSWG) that is also present in the Prx6 from humans and mice. Curiously, the lipase motif is not conserved in all 1-Cys Prxs, and a deeper analysis of the evolution and function of 1-Cys Prxs is in progress. However, the lipase motif is not required for virulence in the mice macrophage and lung models we used here, as a point mutation in the catalytic cysteine was sufficient to abrogate the virulence to the same extent as when the entire *lsfA* coding region was deleted from the *P. aeruginosa* chromosome. Prx proteins have been implied in several diseases in humans. The 1-Cys Prdx6 has been characterized as a tumor inhibitor because it protects mice and human skin cells against lipid peroxidation [41], and the levels of this protein are lower in papillary thyroid carcinomas than in normal thyroid tissue [42]. Prx6 may also be involved in degenerative neuronal disorders, including Alzheimer's disease and prion diseases [43], [44]. In mice infected i.t. with *P. aeruginosa*, LsfA directly or indirectly downregulates the host innate immune response, enabling the pathogen to spread and colonize other organs, leading to an acute infection that results in death. In the macrophage infection model, LsfA is required for *P. aeruginosa* resistance to clearance, with lower TNF-α production in macrophages infected with the PA14 strain compared with macrophages infected with *lsfA* mutants, suggesting that LsfA plays a role in the PA14 immunomodulatory effect. This immunomodulation seems to be related to the macrophages' oxidative state, which is higher when the macrophages are infected with *lsfA* mutant strains than when they are infected with the wild-type PA14. We also found that NADPH oxidase activity is required for the clearance of the *lsfA* mutant, indicating that LsfA is important for PA14 resistance to ROS generated by macrophages. Other *P. aeruginosa* virulence factors also promote immunomodulation. ExoU, a secreted phospholipase, inhibits caspase-1 activation, which is related to pro-IL-1β maturation [45]. The quinolones HHQ and PQS can also negatively regulate the immune system, reducing NF-κB activation, bacterial clearance and TNF-α and IL-6 production [46]. Nevertheless, this is the first evidence of a Prx exerting an immunomodulatory function, protecting the pathogen against phagocytes, reducing phagocyte activation and leading to increased bacterial virulence. We also found that the oxidation of H_2_DCFDA was higher in macrophages infected with *lsfA* mutant strains than in macrophages infected with PA14. In support to our findings, LPS stimulated macrophages, carrying a knockout of the 2-Cys PrxII gene, released more pro-inflammatory cytokines, including TNF-α and IL-6, than wild-type macrophages. This increase in cytokine release is correlated with higher ROS production by the PrxII knockout macrophages, leading to activation of the MAPK and NF-κB pathways [47]. In this study, we show that the lack of a bacterial Prx seems to have the same effect as the lack of PrxII in macrophages, and we speculate that a balance between ROS production and turnover might be necessary to allow the outcome of a host-pathogen interaction to swing from an efficient immune response to virulence, favoring either the host or the bacteria.

In addition to bactericidal activity, ROS can also act as signaling molecules, but the mechanisms involved are poorly understood. Further studies are required to investigate how oxidative stress leads to NF-κB and MAPK activation. However, it appears that bacterial antioxidants play a role in protecting microorganisms from oxidative insult [38] and subvert signaling pathways such as those involved in the immunological response [48].

With the increasing resistance of pathogens to antibiotics, it is crucial to explore new paradigms to develop novel anti-infective drugs, taking advantage of a deeper understanding of bacterial virulence and host defense mechanisms. One aspect that may be explored to achieve this goal is the ROS sensing and production that is employed by both pathogens and hosts. Understanding the role of LsfA in the *P. aeruginosa* immunomodulatory effect may lead to novel therapeutics to overcome the effects of infection by targeting LsfA itself or by improving the host immune response.

## Methods

### Bacterial strains, plasmids, oligonucleotides and culture conditions

All of the strains and plasmids used in this study are listed in Supporting Table S1. The *P. aeruginosa* strains were grown at 37°C in LB broth. The *E. coli* strains were grown in LB supplemented with 100 µg/mL of ampicillin, 50 µg/mL of kanamycin, or 10 µg/mL of gentamicin, when required. The *P. aeruginosa* strains were grown in 250 µg/mL of kanamycin, 20 µg/mL nalidixic acid or 30 µg/mL of gentamicin, when required.

To construct the unmarked in-frame deletion of *lsfA*, primers flanking the upstream and downstream regions of *lsfA* were designed. Amplicons were cloned into pNPTS138 at the *Hind*III and *Eco*RI sites to generate pNPTS138Δ*lsfA*. The resulting construct was used to introduce the *lsfA* deletion into the wild-type PA14 genome by homologous recombination [49], resulting in the Δ*lsfA* mutant, that contains only the first eight N-terminal aminoacids and 31 aminoacids at the LsfA C-terminus. No polar effect is expected, because *lsfA* coding region is distant 249 bp from the next open reading frame in the PA14 genome and the frame of translation was maintained.

The oligonucleotide-directed mutagenesis of the LsfA Cys45 to Ala was performed using the primer pairs listed in Supporting Table S2, and a two-step procedure was performed as previously described [50]. The resulting amplicon was cloned into pNPTS138 and introduced into the PA14 genome by homologous recombination. Mutant clones were screened by PCR followed by digestion or direct sequencing. Again, no polar effects are anticipated, because only one codon was changed.

To construct the *lsfA* complementation strains, the *lsfA* coding region was amplified by PCR using the primer pairs listed in the Supporting Table S2. The resulting amplicon was cloned into pJN105 at the *Eco*RI and *Spe*I sites to generate pLsfA. pLsfA and pJN105 plasmids were introduced into the Δ*lsfA* and the C45A mutants, generating the Δ*lsfA*/pJN105, Δ*lsfA*/pLsfA, C45A/pJN105 and C45A/pLsfA strains.

### Protein expression and purification

The *lsfA* or *lsfAC45A* coding regions were cloned into pProEX-Hta to overexpress His-LsfA or His-LsfAC45A in *E. coli* BL21. Briefly, *E. coli* cultures were grown in 250 mL of LB at 37°C until the culture reached an OD_600 nm_ of 0.5. IPTG was added to a final concentration of 0.6 mM, and the cultures were grown at 30°C for 6 h. Cells were harvested by centrifugation and resuspended in 25 mL lysis buffer (20 mM sodium phosphate pH 7.4, 500 mM NaCl, 20 mM imidazole, 1 mM PMSF). Cell suspensions were lysed by ten 15 second sonication cycles in an ice bath. The lysate was centrifuged at 16000 *g* for 20 min at 4°C. The recombinant proteins His-LsfA and His-LsfAC45A were purified using an Ni-NTA column (Invitrogen) equilibrated with lysis buffer and eluted with an imidazole gradient (20–1000 mM). The eluted fractions were analyzed by SDS-PAGE and pooled, and the buffer was exchanged using a PD-10 column (GE) equilibrated with incomplete reaction buffer (20 mM sodium phosphate pH 7.4, 500 mM NaCl). The proteins were concentrated with Centriprep-Ultracel YM-10.000 MWCO (Millipore). The protein concentration was determined by UV spectroscopy using an extinction coefficient of 33920 M^−1^ cm^−1^, calculated as described by Gill and von Hippel [51], and confirmed using the Bradford reagent (Sigma-Aldrich) according to the manufacturer's protocol.

### Peroxidase activity determination

The *in vitro* peroxidase activity was determined using the ferric-thiocyanate assay [15]. Briefly, 10 µM of purified recombinant protein was incubated at 37°C in 100 µL of reaction buffer (20 mM sodium phosphate pH 7.4, 500 mM NaCl, 1 mM DTT, 10 µM DTPA, 10 µM sodium azide) in the presence or absence of 200 µM H_2_O_2_. At the times indicated, the reaction was stopped by the addition of 20 µL of 2 M HCl and incubated for 10 min at 37°C. Then, 100 µL of 2.5 M KSCN, 100 µL of 20 mM FeSO_4_ and 680 µL of H_2_O were added to the reaction. The absorbance was read at 480 nm. As controls, we used the same conditions in the absence of DTT or without recombinant protein. After the reaction times, the H_2_O_2_ concentration was determined by comparison with a standard curve with different H_2_O_2_ concentrations (15.625–1000 µM).

### Disk diffusion halo assays

Halo inhibition assays were performed as described before with modifications [52]. Cultures of *P. aeruginosa* PA14 and the Δ*lsfA* and C45A mutant and complemented strains were grown overnight in LB broth. Cultures were diluted into fresh LB broth to an OD_600 nm_ of 0.1 and grown to an OD_600 nm_ of 1.0. Plate assays were performed by adding 200 µL of a cell culture to 3 mL of 0.7% LB soft agar. The agar suspensions were spread on LB plates. Sterile paper disks (6 mm in diameter) were saturated with 10 µL of 2.5% hydrogen peroxide and placed on the plates, which were incubated for 16 hours at 37°C. For the complementation assays, 30 ug/mL gentamicin (to maintain the plasmids) and 0.2% arabinose (to induce expression from the *ara* promoter) were added into the LB.

### Cell culture

The macrophage cell line J774 was maintained in R-10 (RPMI 1640 supplemented with 2 mM glutamine, 10% fetal bovine serum (FBS) and 40 µg/mL gentamicin) at 37°C in 5% CO_2_. Macrophages were counted using a Neubauer chamber, and dead cells were excluded by the trypan blue exclusion assay. Macrophages were seeded at 1×10^5^ cells/well (96-well plates) or 2×10^6^ (6-well plates) in R-10 without antibiotic and primed overnight with 10 ng/mL IFN-γ at 37°C in 5% CO_2_.

### *In vitro* infection experiments

PA14 and the Δ*lsfA* and C45A mutant strains were grown in LB broth to an OD_600 nm_ of 2.0. The bacteria were diluted in R-10 without antibiotics. Macrophages that had been previously seeded in 96-well plates were infected at a multiplicity of infection (MOI) of 10. After 1 hour of infection, the supernatant was removed and 200 µg/mL of gentamicin was added to cell cultures to kill extracellular bacteria. After 30 minutes the cells were washed once with PBS and fresh media were added. This point was defined as time 0 (t = 0 h). To determine the numbers of extracellular bacteria, the supernatant was collected, serially diluted, the cells were plated and the CFU were enumerated. To access intracellular bacteria, macrophages were lysed with PBS+0.1% Triton X-100, the lysates were serially diluted, and the CFU were determined. Cytotoxicity was measured for samples taken at the indicated times by quantifying the release of lactate dehydrogenase (LDH) using the CytoTox 96® Non-Radioactive Cytotoxicity Assay (Promega) following the manufacturer's instructions. For NADPH oxidase inhibition, macrophages were treated with 10 µM DPI (diphenylene iodonium) 4 hours before infection and the same steps described above were performed. To quantify TNF-α, macrophages were seeded in six-well plates and infected at an MOI of 10. For inhibition experiments, where indicated, cells were pretreated for 4 hours before infection with an NF-κB inhibitor (10 µM BAY11-7085) or MAPK inhibitors of ERK1/2 (1 µM U0126), p38 (1 µM SB203580), or JNK (1 µM SP600125); alternatively, the cells were treated with 2 mM NAC. At 3 hours post-infection, the supernatants were removed, centrifuged and stored at −20°C. Cytokine quantification was performed by ELISA (R&D systems) following the manufacturer's instructions.

### Oxidative state evaluation

The macrophage oxidative state was determined as described previously [53]. Briefly, macrophages were pre-treated with or without 2 mM NAC for 4 hours before infection. Macrophages were infected with PA14 or with the Δ*lsfA*, C45A or *gacA::tn* mutant strains at an MOI of 10. After the indicated time in post-infection culture medium, the cells were washed with PBS and then incubated with H_2_DCFDA (Invitrogen) at a 2.5 µM final concentration for 30 min at 37°C. Cells were washed with warmed PBS (37°C), resuspended in cold PBS containing 1% FBS and analyzed by fluorescence-activated cell sorting (FACS). Unstained controls were treated similarly. For the baseline fluorescence control, macrophages were uninfected but stained according to the above procedure. The mean fluorescence intensity values were calculated by dividing the values of the infected macrophages by those of the uninfected control.

### Ethics statement

The animal experiments were performed in agreement with the Ethical Principles in Animal Research adopted by the Conselho Nacional de Controle da Experimentação Animal (CONCEA) and in accordance with the recommendations in the Guide for the Care and Use of Laboratory Animals of the National Institutes of Health. The protocol was approved by the Internal Animal Care and Use Committee of the Instituto de Química, Universidade de São Paulo (N°08/2012).

### Animals

Female BALB/c mice (8–12 weeks old) were obtained from the in-house animal facility (Biotério de Produção e Experimentação da Faculdade de Ciências Farmacêuticas e do Instituto de Química da Universidade de São Paulo). Mice were kept on a 12/12-h light/dark cycle with free access to food and water and were maintained under specific pathogen-free conditions. All mice were euthanized in CO_2_ chamber, and every effort was made to minimize suffering.

### Animal inoculation with bacteria

PA14 and the Δ*lsfA* and C45A mutant strains were used for i.t. inoculation as described before [54], with few modifications. Bacteria were grown as described above, harvested by centrifugation at 12000 *g* for 2 min, washed twice in sterile PBS and resuspended in PBS at a concentration of 2×10^6^ bacteria. The CFU/mL were validated by plating serial dilutions of the suspensions. Each mouse received 60 µL of a bacterial suspension. A ketamine/xylazine mixture was injected i.p. to anesthetize the mice before surgery. A midventral incision was made, and the trachea was exposed. The bacterial suspension was inoculated i.t. Controls were inoculated i.t. with 60 µL sterile PBS.

### *In vivo* CFU determination

At 24 hours after infection, the lungs, spleen and liver were harvested for CFU and cytokine measurements. The tissues were homogenized in 1 mL PBS for the lung and spleen and in 2 mL PBS for the liver. The supernatants were collected, and the CFUs were assayed by serial dilution and plating on LB plates. For cytokine measurements, the lung tissues were homogenized, and the supernatants were centrifuged at 12000 *g* for 10 min at 4°C. The cytokines TNF-α, IFN-γ and IL-10 were quantified by ELISA (R&D systems), following the manufacturer's instructions.

### FACS

At 24 hours after infection, the lungs were harvested, minced and digested with collagenase for 30 min at 37°C. The RBCs were lysed by adding NH_4_Cl lysing buffer. The cells were resuspended in PBS with 3% FBS and stained with different combinations of conjugated antibodies, including F4/80-PECy5 (BM8), CD11c-FITC (HL3), CD11b-PE (M1/70) and Ly6G/Ly6C-APC (RB6-8C5), followed by incubation for 20 min on ice. Finally, the cells were washed and resuspended for flow cytometry analysis. FlowJo software (Tree Star) was used to analyze the data.

### Myeloperoxidase activity assay

The myeloperoxidase activity assay was performed as previously described with a few modifications [55], [56]. The animals were infected as described above, and the lungs were harvested, lysed mechanically in the presence of 50 mM sodium phosphate pH 5.4, 5 mM EDTA and 0.5% cetyltrimethylammonium bromide, ultrasonicated and centrifuged. The supernatant (50 µL) was mixed with an equal volume of 3 mM 3,3′,5,5′-tetramethylbenzidine dihydrochloride (TMB) for 2 minutes. The reaction was stopped by the addition of 25 µL of 2M H_2_SO_4_. The optical density (OD) was measured at 450 nm.

### Survival

After i.t. infection with wild-type PA14 or the Δ*lsfA* or C45A strains, the treated (N = 8) and control (N = 3) groups were observed for survival. All deaths reported were from moribund/euthanized mice. Mice with labored or rapid breathing, decreased motility, ruffled or abnormal-looking fur or other obvious signs of distress were considered to be moribund as described before [57].

### Statistical analyses

Prism 5 (GraphPad Inc.) was used for all statistical analyses. Kaplan-Meier survival curves were plotted, and significance was calculated using the log-rank test. The data were compared using the one-way or two-way analysis of variance (ANOVA) followed by Bonferroni's multiple comparison test.

## Supporting Information

Figure S1 **Multiple sequence alignment of 1-Cys Prxs.** ClustalW alignment of 1-Cys Prxs from different species. The conserved Prx6 motifs are boxed; the catalytic Cys is highlighted in green, and the lipase motif in blue. Sequences were obtained from GenBank. The GI numbers are: GI:116051470, GI:78065379, GI:107021887, GI:53720358, GI:4758638, GI:3219774, GI:82540481, GI:160877634, GI:6319407, GI:85093072, GI:70983971, GI:152986639, GI:161525740, GI:33592121, GI:15598646, GI:431800278, GI:104779502 and GI:50285063.
(DOCX)

Figure S2 **Lack of LsfA does not impair bacterial growth in liquid cultures and in biofilms.** PA14 or the Δ*lsfA* or C45A mutant strains were grown in LB (**A**) or M63 minimal medium (**B**) at 37°C, and data were recorded in a SpectraMax Paradigm apparatus. Data are representative of triplicate experiments. In (**C**), cultures were grown in LB without shaking in glass tubes for 16 h, the tubes were washed and the adhered cells stained with crystal violet.
(DOCX)

Figure S3 **Complementation of the** ***lsfA*** **and C45A sensitivity to H_2_O_2_.** Plasmids pJN105 (control) or pLsfA (pJN105 containing the wild-type *lsfA* gene under an arabinose inducible promoter) were introduced in the *lsfA* and C45A mutant strains and the resulting clones were seeded on LB containing 0.2% arabinose and 30 µg/mL gentamicin. Filter discs containing 2.5% H_2_O_2_ were placed on top of the agar, the plates were incubated overnight at 37°C and the haloes were measured. The numbers inside the plates refer to the diameter of the haloes ± SD. The figure shows one representative experiment from duplicate assays.
(DOCX)

Figure S4 **The wild-type strain and** ***lsfA*** **mutants show same levels of macrophages cytotoxicity.** J774 macrophages were incubated with *P. aeruginosa* PA14 or the Δ*lsfA* or C45A mutants at an MOI of 10. At the indicated time points, the supernatants were collected and diluted, the cells were washed with PBS and R-10 containing 200 µg/mL gentamicin was added to the wells for 30 min, cells were washed and incubated in R-10. At the indicated time points, lactate dehydrogenase (LDH) release was determined as a measure of macrophage death. Data are the means ± SD from at least three independent experiments performed in triplicate.
(DOCX)

Figure S5 **NAC reduces macrophage oxidative stress in C45A and PA14-infected macrophages.** Macrophages pre-treated with 2 mM NAC for 4 hours (grey bars) were infected with PA14 or with the C45A mutant strains at an MOI of 10. A control group was not incubated with NAC (white bars). After the indicated times post-infection, the cells were washed with PBS, incubated with 2.5 µM H_2_DCFDA for 30 min at 37°C, washed again with warmed PBS (37°C), resuspended in cold PBS containing 1% FBS and analyzed by fluorescence-activated cell sorting (FACS). Unstained controls were treated similarly. For the baseline fluorescence control, macrophages were uninfected but stained. The mean fluorescence intensity values were calculated by dividing the values of the infected macrophages by those of the uninfected control.
(DOCX)

Figure S6 **IL-10 production in** ***P. aeruginosa*** **-infected mice is not affected by a mutation in** ***lsfA*** **.** BALB/c mice were infected i.t. with 2×10^6^ bacteria of the wild-type strain PA14 or the C45A mutant. The infected animals and controls (n = 3) were sacrificed 24 hours post-infection, the lungs were macerated, and the IL-10 concentrations were determined by ELISA. Data are the means ± SD from at least three independent experiments performed in triplicate.
(DOCX)

Table S1 **Strains and plasmids used in this study.**
(DOCX)

Table S2 **Oligonucleotides used in this work.**
(DOCX)
