# Supplementary material for: Involvement of a 1-Cys Peroxiredoxin in Bacterial Virulence
Source: PLoS Pathog. 2014 Oct 16;10(10):e1004442. doi: 10.1371/journal.ppat.1004442 (PMC4199769; doi:10.1371/journal.ppat.1004442)
Supplement: Figure S1 — Multiple sequence alignment of 1-Cys Prxs. ClustalW alignment of 1-Cys Prxs from different species. The conserved Prx6 motifs are boxed; the catalytic Cys is highlighted in green, and the lipase motif in blue. Sequences were obtained from GenBank. The GI numbers are: GI:116051470, GI:78065379, GI:107021887, GI:53720358, GI:4758638, GI:3219774, GI:82540481, GI:160877634, GI:6319407, GI:85093072, GI:70983971, GI:152986639, GI:161525740, GI:33592121, GI:15598646, GI:431800278, GI:104779502 and GI:50285063. (DOCX) [file ppat.1004442.s001.docx]

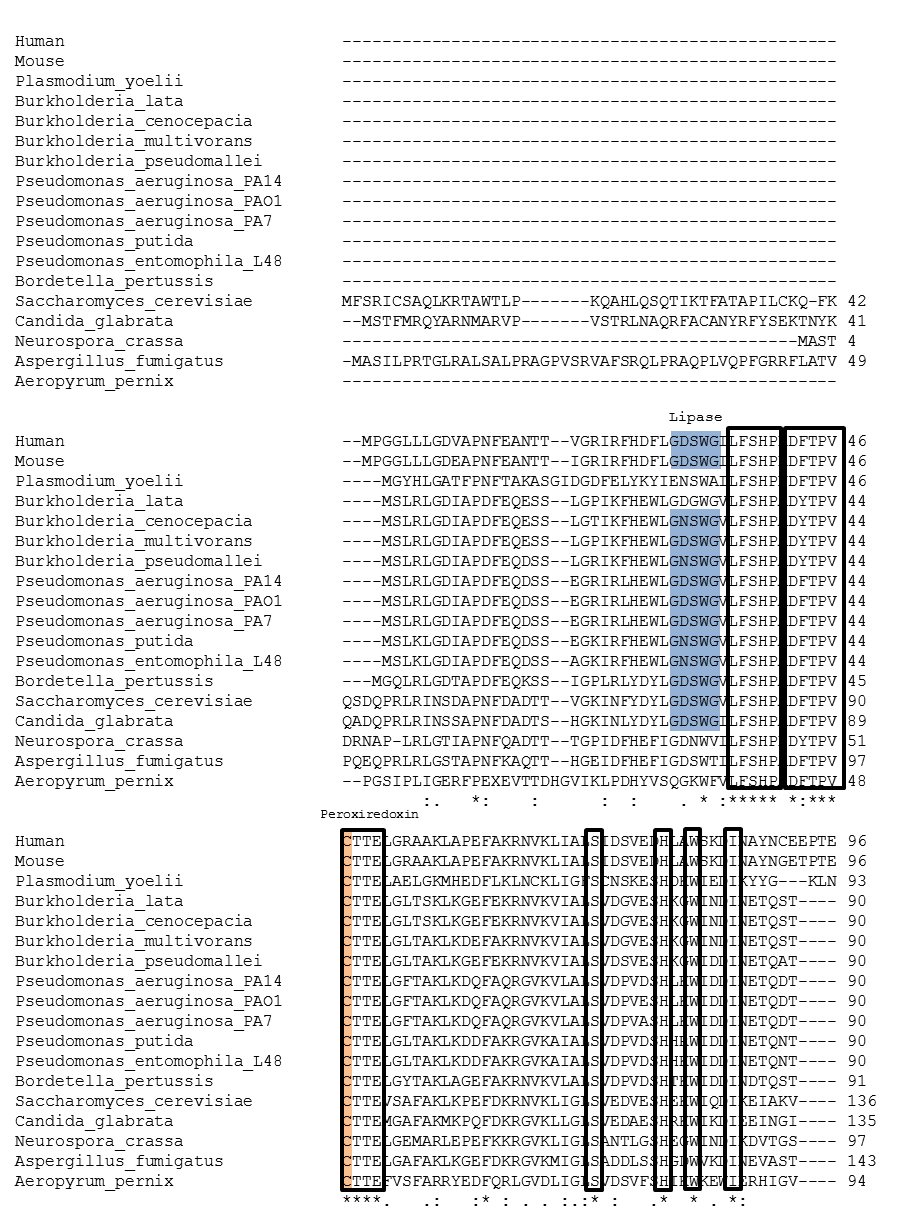


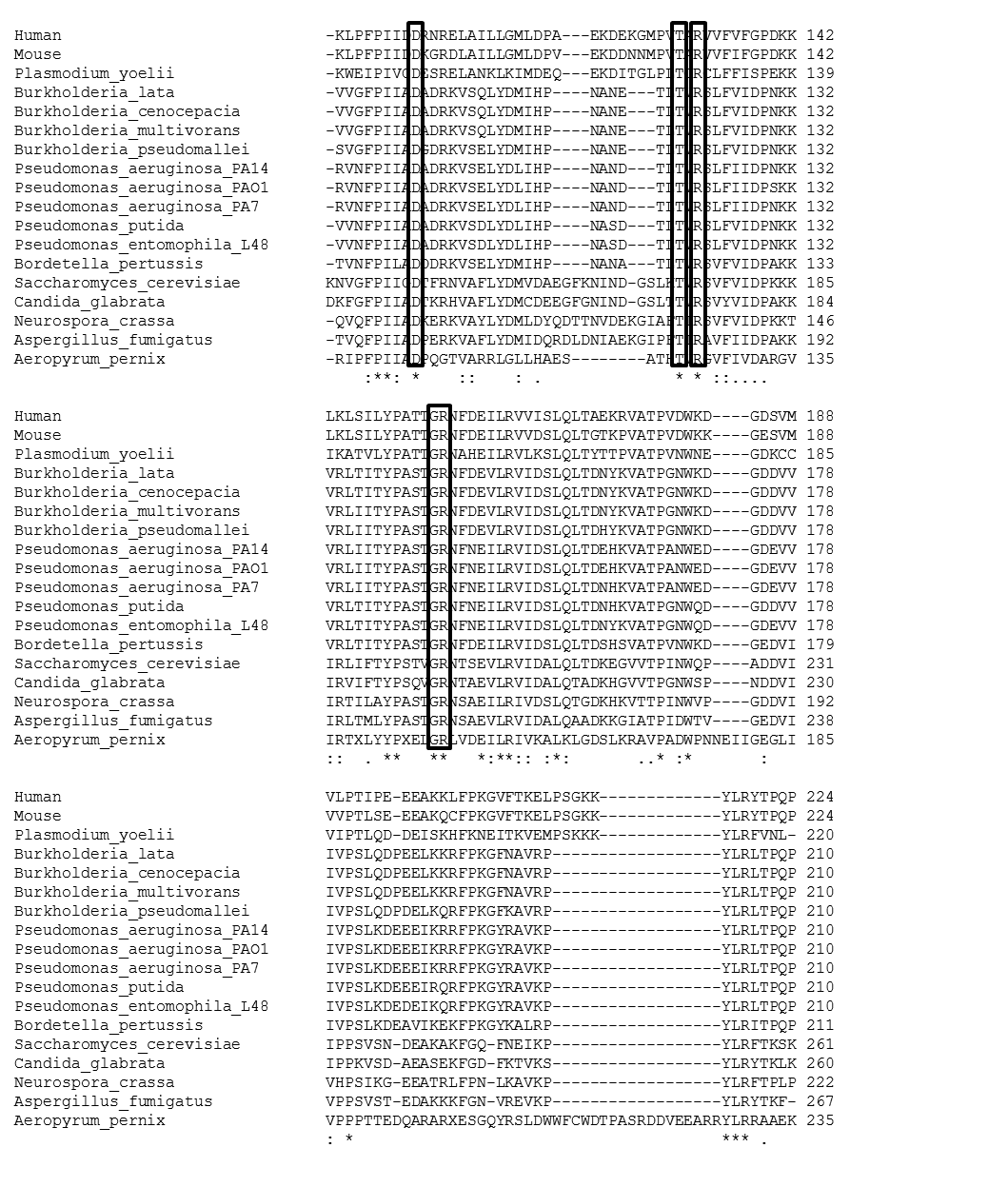


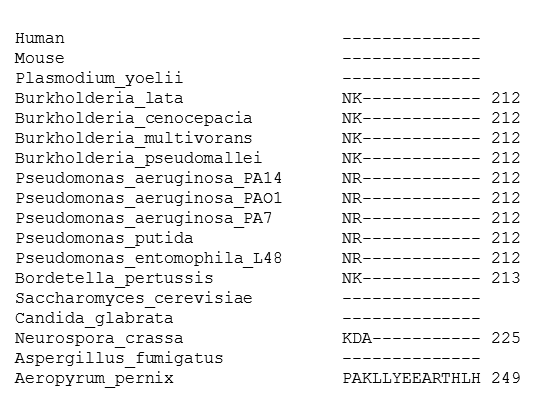


**Figure S1. Multiple sequence alignment of 1-Cys Prxs.** ClustalW alignment of 1-Cys Prxs from different species. The conserved Prx6 motifs are boxed; the catalytic Cys is highlighted in green, and the lipase motif in blue. Sequences were obtained from GenBank. The GI numbers are: GI:116051470, GI:78065379, GI:107021887, GI:53720358, GI:4758638, GI:3219774, GI:82540481, GI:160877634, GI:6319407, GI:85093072, GI:70983971, GI:152986639, GI:161525740, GI:33592121, GI:15598646, GI:431800278, GI:104779502 and GI:50285063.
