# Supplementary material for: Involvement of a 1-Cys Peroxiredoxin in Bacterial Virulence
Source: PLoS Pathog. 2014 Oct 16;10(10):e1004442. doi: 10.1371/journal.ppat.1004442 (PMC4199769; doi:10.1371/journal.ppat.1004442)
Supplement: Figure S2 — Lack of LsfA does not impair bacterial growth in liquid cultures and in biofilms. PA14 or the ΔlsfA or C45A mutant strains were grown in LB (A) or M63 minimal medium (B) at 37°C, and data were recorded in a SpectraMax Paradigm apparatus. Data are representative of triplicate experiments. In (C), cultures were grown in LB without shaking in glass tubes for 16 h, the tubes were washed and the adhered cells stained with crystal violet. (DOCX) [file ppat.1004442.s002.docx]

**
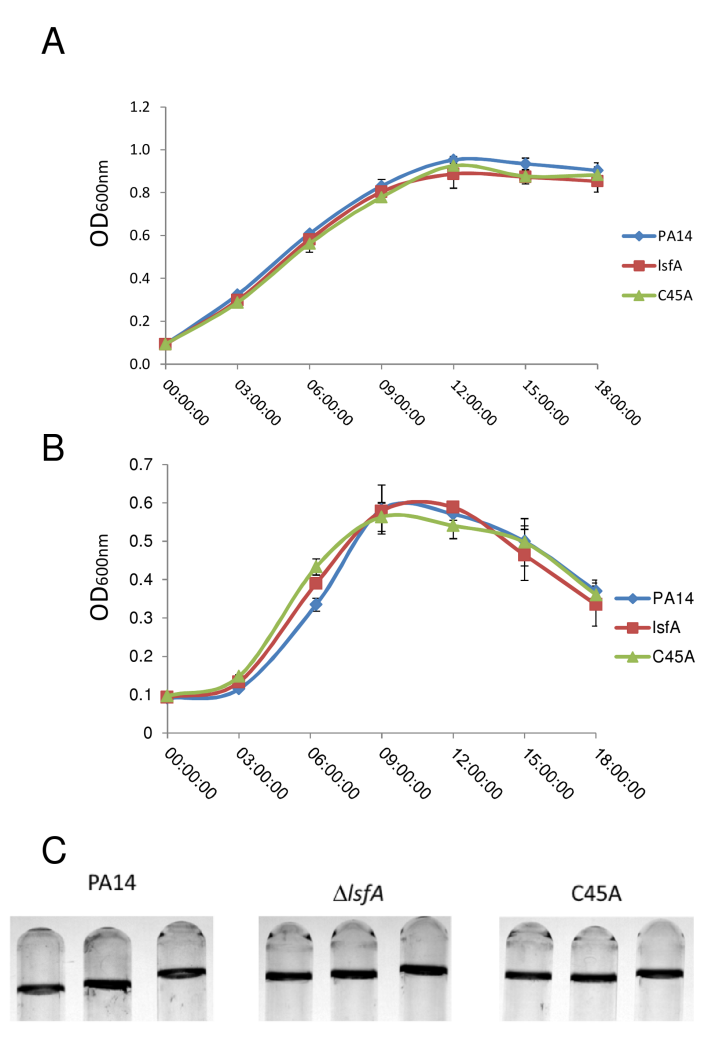
**

**Figure S2. Lack of LsfA does not impair bacterial growth.** PA14 or the ∆*lsfA* or C45A mutant strains were grown in LB **(A)** or M63 **(B)** at 37°C, and data were recorded in a SpectraMax Paradigm apparatus. Data are representative of triplicate experiments.
