# Supplementary material for: Involvement of a 1-Cys Peroxiredoxin in Bacterial Virulence
Source: PLoS Pathog. 2014 Oct 16;10(10):e1004442. doi: 10.1371/journal.ppat.1004442 (PMC4199769; doi:10.1371/journal.ppat.1004442)
Supplement: Figure S3 — Complementation of the lsfA and C45A sensitivity to H2O2. Plasmids pJN105 (control) or pLsfA (pJN105 containing the wild-type lsfA gene under an arabinose inducible promoter) were introduced in the lsfA and C45A mutant strains and the resulting clones were seeded on LB containing 0.2% arabinose and 30 µg/mL gentamicin. Filter discs containing 2.5% H2O2 were placed on top of the agar, the plates were incubated overnight at 37°C and the haloes were measured. The numbers inside the plates refer to the diameter of the haloes ± SD. The figure shows one representative experiment from duplicate assays. (DOCX) [file ppat.1004442.s003.docx]

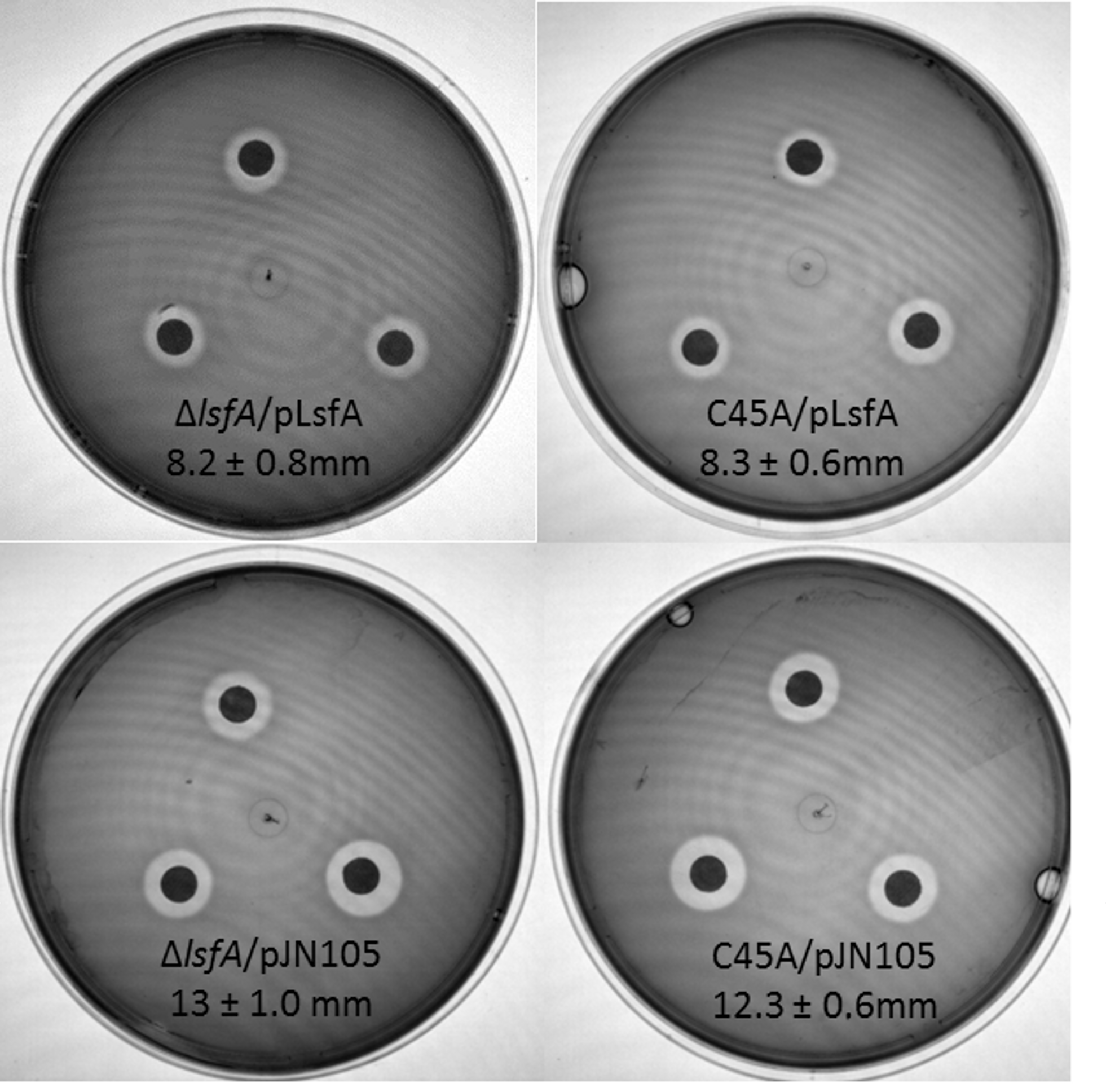


**Figure S3. Complementation of the Δ*lsfA* and C45A sensitivity to H_2_O_2_**. Plasmids pJN105 (control) or pLsfA (pJN105 containing the wild-type *lsfA* gene under an arabinose inducible promoter) were introduced in the Δ*lsfA* and C45A mutant strains and the resulting clones were seeded on LB containing 0.2% arabinose and 30 μg/mL gentamicin. Filter discs containing 2.5% H_2_O_2_ were placed on top of the agar, the plates were incubated overnight at 37^o^C and the haloes were measured. The numbers inside the plates refer to the diameter of the haloes ± SD. The figure shows one representative experiment from duplicate assays.
