# Supplementary material for: Involvement of a 1-Cys Peroxiredoxin in Bacterial Virulence
Source: PLoS Pathog. 2014 Oct 16;10(10):e1004442. doi: 10.1371/journal.ppat.1004442 (PMC4199769; doi:10.1371/journal.ppat.1004442)
Supplement: Figure S4 — The wild-type strain and lsfA mutants show same levels of macrophages cytotoxicity. J774 macrophages were incubated with P. aeruginosa PA14 or the ΔlsfA or C45A mutants at an MOI of 10. At the indicated time points, the supernatants were collected and diluted, the cells were washed with PBS and R-10 containing 200 µg/mL gentamicin was added to the wells for 30 min, cells were washed and incubated in R-10. At the indicated time points, lactate dehydrogenase (LDH) release was determined as a measure of macrophage death. Data are the means ± SD from at least three independent experiments performed in triplicate. (DOCX) [file ppat.1004442.s004.docx]

**Figure S4.** **The wild-type strain and *lsfA* mutants show same levels of macrophages
cytotoxicity.** J774 macrophages were incubated with *P. aeruginosa* PA14 or the ∆*lsfA* or C45A mutants at an MOI of 10. At the indicated time points, the supernatants were collected and diluted, the cells were washed with PBS and R-10 containing 200 μg/mL gentamicin was added to the wells for 30 min, cells were washed and incubated in R10. At the indicated time points, lactate dehydrogenase (LDH) release was determined as a measure of macrophage death. Data are the means ± SD from at least three independent experiments performed in triplicate.
