# Supplementary material for: Involvement of a 1-Cys Peroxiredoxin in Bacterial Virulence
Source: PLoS Pathog. 2014 Oct 16;10(10):e1004442. doi: 10.1371/journal.ppat.1004442 (PMC4199769; doi:10.1371/journal.ppat.1004442)
Supplement: Figure S5 — NAC reduces macrophage oxidative stress in C45A and PA14-infected macrophages. Macrophages pre-treated with 2 mM NAC for 4 hours (grey bars) were infected with PA14 or with the C45A mutant strains at an MOI of 10. A control group was not incubated with NAC (white bars). After the indicated times post-infection, the cells were washed with PBS, incubated with 2.5 µM H2DCFDA for 30 min at 37°C, washed again with warmed PBS (37°C), resuspended in cold PBS containing 1% FBS and analyzed by fluorescence-activated cell sorting (FACS). Unstained controls were treated similarly. For the baseline fluorescence control, macrophages were uninfected but stained. The mean fluorescence intensity values were calculated by dividing the values of the infected macrophages by those of the uninfected control. (DOCX) [file ppat.1004442.s005.docx]

**

**

**Figure S5. NAC reduces macrophage oxidative stress in C45A and PA14-infected macrophages.** Macrophages pre-treated with 2 mM NAC for 4 hours (grey bars) were infected with PA14 or with the C45A mutant strains at an MOI of 10. A control group was not incubated with NAC (white bars). After the indicated times post-infection, the cells were washed with PBS, incubated with 2.5 μM H_2_DCFDA for 30 min at 37°C, washed again with warmed PBS (37°C), resuspended in cold PBS containing 1% FBS and analyzed by fluorescence-activated cell sorting (FACS). Unstained controls were treated similarly. For the baseline fluorescence control, macrophages were uninfected but stained. The mean fluorescence intensity values were calculated by dividing the values of the infected macrophages by those of the uninfected control.
