# Supplementary material for: Involvement of a 1-Cys Peroxiredoxin in Bacterial Virulence
Source: PLoS Pathog. 2014 Oct 16;10(10):e1004442. doi: 10.1371/journal.ppat.1004442 (PMC4199769; doi:10.1371/journal.ppat.1004442)
Supplement: Figure S6 — IL-10 production in P. aeruginosa -infected mice is not affected by a mutation in lsfA . BALB/c mice were infected i.t. with 2×10^6^ bacteria of the wild-type strain PA14 or the C45A mutant. The infected animals and controls (n = 3) were sacrificed 24 hours post-infection, the lungs were macerated, and the IL-10 concentrations were determined by ELISA. Data are the means ± SD from at least three independent experiments performed in triplicate. (DOCX) [file ppat.1004442.s006.docx]

**

**

**Figure S6. IL-10 production in *P. aeruginosa*-infected mice is not affected by a mutation in *lsfA***. BALB/c mice were infected i.t. with 2 x 10^6^ bacteria of the wild-type strain PA14 or the C45A mutant. The infected animals and controls (n=3) were sacrificed 24 hours post-infection, the lungs were macerated, and the IL-10 concentrations were determined by ELISA. Data are the means ± SD from at least three independent experiments performed in triplicate.
