# Supplementary material for: Involvement of a 1-Cys Peroxiredoxin in Bacterial Virulence
Source: PLoS Pathog. 2014 Oct 16;10(10):e1004442. doi: 10.1371/journal.ppat.1004442 (PMC4199769; doi:10.1371/journal.ppat.1004442)
Supplement: Table S1 — Strains and plasmids used in this study. (DOCX) [file ppat.1004442.s007.docx]

**Table S1.-Strains and Plasmids used in this study**

| **Strains** | **Description** | **Reference** |
| --- | --- | --- |
| ***P. aeruginosa*** |  |  |
| PA14 | Wild-type *P. aeruginosa* UCBPP-PA14 | (Rahme et al., 1995) |
| *gacA* | PA14 with a *mar7* transposon inserted in the *gacA* gene | (Liberati et al., 2006) |
| Δ*lsfA* | PA14 with deletion in *lsfA* | This Work |
| C45A | PA14 with point mutation in *lsfA* in Cys45Ala | This Work |
| Δ*lsfA*/pJN105 | Δ*lsfA* harboring pJN105 plasmid | This Work |
| Δ*lsfA*/pLsfA | Δ*lsfA* harboring pLsfA plasmid | This Work |
| C45A/pJN105 | C45A harboring pJN105 plasmid | This Work |
| C45A/pLsfA | C45A harboring pLsfA plasmid | This Work |
| ***E. coli*** |  |  |
| DH5α | *supE44 lacU169* (80 *lacZ*M15) *hsdR17* *recA1 endA11 gyrA96 thi-1 relA1* | Invitrogen |
| S17-1 | *prothirecA* *hsd*R (r ^-^ m^+^) Tp^r^ Sm^r^ Km^s^ [∧ RP4-2-Tc::Um-Km::Tn7] | (Simon et al., 1983) |
| BL21(DE3) | F-*ompTgaldcmlon* hsdS_b_(r_b_^-^m_b_^-^)⎣(DE3 [*lacI lac*UV5-T7 gene 1 *ind*1 *sam*7 *nin*5]) | (Studier and Moffatt, 1986) |
| RB303 | *E. coli* BL21(DE3) carrying pGHK005 | This work |
| RB304 | *E. coli* BL21(DE3) carrying pGHK006 | This work |
| **Macrophage** |  |  |
| J774.A1 | Monocyte/Macrophage cell line | (Snyderman et al., 1977) |
| **Plasmid** | **Description** | **Reference** |
| pGEM-T easy | Cloning vector; Apr | Promega |
| pNPTS138 | Replicon ColE1,oriT, *npt*(Km^r^), *sacB* | (Tsai and Alley, 2000) |
| pProEx HTa | Expression vector. Ap^r^ | Invitrogen |
| pJN105 | araC-pBAD cloned in pBBR1 MCS-5; GmR | (Newman and Fuqua, 1999) |
| pLsfA | *lsfA* coding region cloned in pJN105 | This work |
| pGHK001 | 729 bp upstream of *lsfA* coding region cloned in pGEM-T Easy | This work |
| pGHK002 | 799 bp downstream of *lsfA* coding region cloned in pGEM-T Easy | This work |
| pGHK003 | 729 bp upstream and 799 downstream of *lsfA* coding region cloned in pNPTS138 | This work |
| pGHK004 | 2047 bp region containg a point mutation in *lsfAC45A* cloned in pNPTS138 | This work |
| pGHK005 | *lsfA* coding region cloned in pProEx HTa | This work |
| pGHK006 | *lsfAC45A* coding region cloned in pProEx HTa | This work |

**Supplementary References for Table S1**

Liberati, NT, Urbach, JM, Miyata, S, Lee, DG, Drenkard, E et al. (2006) An ordered, nonredundant library of *Pseudomonas aeruginosa* strain PA14 transposon insertion mutants. Proc Natl Acad Sci USA *103*, 2833-2838.

Newman JR, Fuqua C (1999) Broad-host-range expression vectors that carry the L-arabinose-inducible *Escherichia coli* *araBAD* promoter and the *araC* regulator. Gene 227: 197-203.

Rahme, LG, Stevens, EJ, Wolfort, SF, Shao, J, Tompkins, RG, and Ausubel, FM (1995). Common virulence factors for bacterial pathogenicity in plants and animals. Science *268*, 1899-1902.

Snyderman, RMCP, Fischer, DG, Koren, HS (1977). Biologic and biochemical activities of continuous macrophage cell lines P388D1 and J774.1. J Immunol *119*, 2060 - 2066.

Simon, R, Priefer, U, and Puhler, A (1983). A broad host range mobilization system for *in vivo* genetic engineering: transposon mutagenesis in gram negative bacteria. Biotech (NY) *1*, 784-790.

Studier, FW, and Moffatt, BA (1986). Use of bacteriophage T7 RNA polymerase to direct selective high-level expression of cloned genes. J Mol Biol *189*, 113-130.

Tsai, JW, and Alley, MR (2000). Proteolysis of the McpA chemoreceptor does not require the *Caulobacter* major chemotaxis operon. J Bacteriol *182*, 504-507.
