# Supplementary material for: Involvement of a 1-Cys Peroxiredoxin in Bacterial Virulence
Source: PLoS Pathog. 2014 Oct 16;10(10):e1004442. doi: 10.1371/journal.ppat.1004442 (PMC4199769; doi:10.1371/journal.ppat.1004442)
Supplement: Table S2 — Oligonucleotides used in this work. (DOCX) [file ppat.1004442.s008.docx]

**Table S2. Oligonucleotides used in this work**

| Primer | Sequence^1,2^ | Use |
| --- | --- | --- |
| Del_LsfA 1 *Hin*DIII | **AAGCTT**CACGTCGACCGGTTTTTC | *lsfA deletion* |
| Del_Lsfa 1 *Bam*HI | **GGATCC**GATGTCGCCGAGTCTGAGG |  |
| Del_LsfA 2 *Bam*HI | **GGATCC**TCGCTGAAGGACGAGGAAG | *lsfA* deletion |
| Del_LsfA 2 *Eco*RI | **GAATTC**GCGGAGCAGGAACAGGTA |  |
| LsfA_C45A left | ACCCCGGTGGCAACCACCGAG | site directed mutagenesis in *lsfA* |
| LsfA_C45A right | CTCGGTGGTTGCCACCGGGGT |  |
| Overexp LsfA Left | **GAATTC**CTCAGACTCGGCGACATC | *lsfA* coding region, for pProEx cloning |
| Overexp LsfA Right | **AAGCTT**GACGAATCCGGCCTCAG |  |
| LEFT_pJN105_LsfA | **GAATTC**TGGGTTCCAACACGAACA | *lsfA* coding region, for pJN105 cloning |
| RIGHT_pJN105_LsfA | **ACTAGT**GACGAATCCGGCCTCAG |  |

^1^Restriction site are in bold

^2^Underlined bases were used to generate site-direct mutations
